# Prediction of cancer survival for cohorts of patients most recently diagnosed using multi-model inference

**DOI:** 10.1177/0962280220934501

**Published:** 2020-10-06

**Authors:** Camille Maringe, Aurélien Belot, Bernard Rachet

**Affiliations:** Department of Non-Communicable Disease Epidemiology, London School of Hygiene & Tropical Medicine, Keppel Street, London, UK

**Keywords:** Cancer survival, prediction, projection, multi-model inference, Akaike information criteria, Bayesian information criteria

## Abstract

Despite a large choice of models, functional forms and types of effects, the selection of excess hazard models for prediction of population cancer survival is not widespread in the literature. We propose multi-model inference based on excess hazard model(s) selected using Akaike information criteria or Bayesian information criteria for prediction and projection of cancer survival. We evaluate the properties of this approach using empirical data of patients diagnosed with breast, colon or lung cancer in 1990–2011. We artificially censor the data on 31 December 2010 and predict five-year survival for the 2010 and 2011 cohorts. We compare these predictions to the observed five-year cohort estimates of cancer survival and contrast them to predictions from an a priori selected simple model, and from the period approach. We illustrate the approach by replicating it for cohorts of patients for which stage at diagnosis and other important prognosis factors are available. We find that model-averaged predictions and projections of survival have close to minimal differences with the Pohar-Perme estimation of survival in many instances, particularly in subgroups of the population. Advantages of information-criterion based model selection include (i) transparent model-building strategy, (ii) accounting for model selection uncertainty, (iii) no a priori assumption for effects, and (iv) projections for patients outside of the sample.

## 1 Introduction

Cancer survival is a public health measure that complements the reporting of incidence, prevalence and mortality.^1^ Projections of incidence and mortality figures are common practice.^2–5^ These trends are often extrapolated to get estimates of the future burden of cancer for planning purposes, or based on scenarios reflecting the likely effect of new screening strategies, or changes in the distributions of risk factors.^6–8^

Survival models do not show good predictive performances.^9,10^ This may be one of the reasons why prediction and projection of survival are, by far, less routinely made.

While prognosis research is focused on individual risk prediction scores,^11,12^ we are interested here in predicting cancer survival for cohorts of patients as a whole or by reasonably large sub-groups, and we refer to these as population predictions. In that context, accurate individual-level predictions are less crucial since we intend to produce marginal estimates of survival. Many different survival models may be fitted to the data, and we focus here on regression models assuming multiplicative effects of explanatory variables on the hazard of death. A specificity of survival analysis is that the effects of variables may vary through follow-up time (time-dependent effect) and selecting the right effects can then be challenging. Background knowledge and model selection algorithms help narrow down the choice of models to the most appropriate one(s).^13^

When considered, model selection tends to be based on likelihood ratio tests,^14–16^ using usually backward or forward selection strategy (or a combination of both). A single model is therefore selected as the best fit for the data or for subsequent prediction. We see two drawbacks to such approach. First, it means discarding effects that may have been equally likely to those selected. Second, once a model is chosen, no uncertainty relative to the selection is pertained to the model-based estimates and post-model selection inference.^17,18^ In the context of prediction, we believe it is critical to consider that there may be several models equally likely to have generated the data. This is the philosophy of Bayesian model selection^19^ and multi-model inference also described by Burnham and Anderson.^20^ Lastly, hypothesis testing may perform poorly when using observational data,^20^ as they are designed to detect discrepancies between the model and the data, rather than express how close a model is to the data: the larger the sample, the easier it is to detect (small) discrepancies.^21^

The Akaike Information Criteria (AIC)^22^ is a likelihood-based measure that estimates the expected relative distance between the fitted model and the unknown true mechanism. AIC values can be compared between different, non-necessarily nested, models. Contrasting AIC values asymptotically coincides with generalised leave-one-out cross-validation.^23^ The Bayesian (or Schwarz) Information Criteria (BIC) is an estimator of the Bayes Factor, aiming to quantify the evidence for one model against another.^24^

This article is organised as follows: the next section introduces the cancer registry data linked to electronic health records. The following section discusses the setting of relative survival for the estimation of cancer net survival,^25^ the multi-model inference and the prediction tools used to assess the accuracy of the predicted estimates of net survival. Then, we present results on a historical, low-resolution, data setting for the prediction and projection of five-year survival for patients most recently diagnosed, to highlight the properties of the method. An application follows, based on more recent, high-resolution data including information on stage at diagnosis: a setting that motivates multivariable modelling and multi-model inference. The discussion highlights the advantages of multi-model inference and potential extensions conclude the manuscript.

## 2 Material

We use data of the population-based cancer registry of England. Virtually all cancer cases diagnosed in England are registered. Quality controls are performed at the time of registration, and prior to data analysis^26^ to ensure there are no duplicate registrations and the sequence of dates (birth, diagnosis, latest vital status) is logical, among other checks.

We analyse records of adult patients (15–99 years) diagnosed with malignant lung cancer (men only, ICD-9: 162, ICD-10: C33-C34), breast cancer (women, ICD-9: 174, ICD-10: C50) or colon cancer (men only, ICD-9: 153, ICD-10: C18) in 1990 through to 2011. We define patients’ information on socio-economic status based on their postcode of residence using the Townsend^27^ and the income domain of the Index for Multiple Deprivation^28,29^ scores for the years 1990–2000 and 2001–2011, respectively. Both scores are ecological and based upon responses to census questions relative to income and wealth, by small areas (Enumeration Districts until 2000 and Lower Super Output Areas from 2001). The areas are grouped by quintiles of area-level deprivation distribution, according to their score, from least (quintile 1) to most (quintile 5) deprived.

The latest vital status of patients is obtained from linking the cancer registrations to the mortality databases maintained by the Office for National Statistics. A vital status indicator is assigned to all patients together with a date of last known vital status, or death where appropriate. Patients are followed up until 31 December 2015.

Stage at diagnosis is one of the most important predictors of survival. It is based on the T (tumour size), N (lymph node involvement) and M (metastatic or not) components of the TNM stage at diagnosis classification.^30^ Until recently, its recording, through combining information from pathology laboratories, hospital records, and Multidisciplinary Team records, was not complete or accurate for many cancers in population-based cancer registry data in England. High proportions of missing information on stage at diagnosis make it difficult to study its effect on survival through time.^31^

## 3 Methods

### 3.1 Scenarios studied

#### 3.1.1 Low-resolution data setting: empirical evaluation of the properties of multi-model inference

We focus here on the cancers of colon (men), lung (men), and breast (women). First, we artificially restrict the follow-up to 31 December 2010. To compare the impact that varying numbers of cohorts have on the accuracy of the predictions, we run several model selections on cohorts of patients diagnosed in 1990–2010, 1995–2010, 2000–2010, or 2005–2010. We predict excess hazard and five-year cancer survival for patients diagnosed in 2010, patients for whom only the first year of follow-up contributed to the model selection. We also project excess hazard and five-year cancer survival for patients diagnosed in 2011.

**Figure 1.**
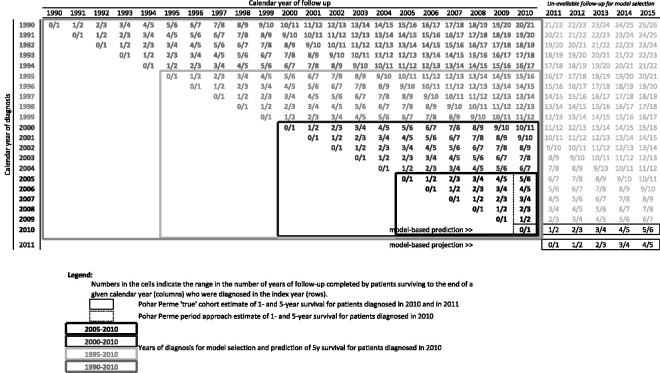
Structure of the data as used in the low-resolution data setting.

Since follow-up beyond 31 December 2010 is neither used in the estimation of the regression parameters nor in model selection, we are able to contrast the predicted five-year survival of these patients to their actual survival as observed until 31 December 2015 by group of patients and overall. Similarly, patients diagnosed in 2011 do not contribute to the modelling at all. Nonetheless we compare the results of their projections to their five-year survival as observed until 31 December 2015. Figure 1 summarizes how the data are used in this low-resolution data setting, highlighting what is supposed known and unknown, and the cohorts of patients used in model selection.

#### 3.1.2 High-resolution data setting: illustration

We identify groups of patients for whom the proportion of missing stage at diagnosis is the lowest. For lung cancer, we select patients who were diagnosed at ages 50–74 between 2008 and 2012, and living in the East and North East of England (missing stage up to 14%).^32^ For breast cancer, we analyse patients diagnosed at ages 50–84 in 2005–2011, living in the West Midlands (stage missing up to 12%).^33,34^ For those two groups of patients, we can develop prediction models that include stage at diagnosis, as well as an indicator of mode of presentation (emergency for lung cancer, screening for breast cancer) and performance status (lung cancer). We predict lung and breast cancer survival up to four years after diagnosis for patients diagnosed in 2010 or 2011, for whom only the first year after diagnosis contributes to model selection and estimation of effects, and project cancer survival for patients diagnosed in 2011 and 2012.

### 3.2 Net survival

We aim to answer the following question: “What is the predicted *cancer* survival of cancer patients?” We focus on net survival, which measures survival among a defined cohort of cancer patients under the assumption that they only die of the studied cancer. This marginal survival measure is therefore independent of the deaths from other causes. Thus, this is the quantity of interest when aiming to compare cancer survival between countries and over time. Despite international classification, the determination of the cause of death is not standardised enough through time, or between registrars, for the cause of death to be used in our analyses. Hence, we aim to estimate cancer (net) survival in the relative survival setting using excess hazard models.^35,36^ Several forms of models exist exhibiting different ways of modelling the baseline excess hazard of death, and interactions with follow-up time.^37–45^

The main assumption of excess hazard models is that the observed mortality of the cohort of patients (λ) is the sum of two forces of mortality: the excess mortality hazard ($\lambda_{E}$), assumed to be the mortality hazard directly or indirectly due to cancer, and the expected or other causes mortality hazard, which is considered to be well approximated by the general population mortality hazard ($\lambda_{P}$).^46,47^

$$\lambda (t,x)=\lambda_{E}\;(t,x)+\lambda_{P}\;(a+t,y+t,z)$$

The cancer mortality hazard,$\;\lambda_{E}$, at time t for given patient’s covariates x, such as age at diagnosis (a) and calendar year of diagnosis (y), is what we need to estimate. We derive mortality due to causes other than cancer at time t by population tables of mortality, defined for the population from which cancer patients come from, i.e. with similar features (age at time t: a+t, calendar year at time t: y+t, sex, levels of deprivation, geographical area of residence, ethnicity when possible, etc., summarised in z, a subset of patient’s covariates x).

First, we use the non-parametric Pohar-Perme (PP) estimator,^48^ a consistent estimator of net survival, to obtain cancer survival for patients diagnosed in 2010 with follow-up until 2015. That estimator relies on the observed and expected proportions of patients still alive at each time of event. Patients may die of other causes, thus preventing their cancer survival time to be observed. The cohort of patients therefore changes structurally throughout follow-up time and is not representative of the original cohort of patients. An inverse-probability-of-censoring weighting is applied to adjust for this informative censoring, so that the contribution of each patient to the estimator is weighted by the inverse of the probability that the patient is expected to survive until each time of event (using population tables of mortality). The period approach PP estimator is also used to predict survival for patients diagnosed in 2010, using information from patients diagnosed in previous cohorts, alive in 2010, with potential follow-up until 31 December 2010 (‘period approach’).^49^ The period approach derives survival in a similar fashion to life expectation.

Second, we use flexible, multivariable models, to estimate excess mortality hazard $\lambda_{E}(t,x)$, individual ($S_{E,i}\left(t,x\right)$) and cohort ($S_{E}(t)$) net survival.^50^ The logarithm of the baseline excess hazard is modelled using restricted cubic spline functions, with three degrees of freedom, that is two internal knots (located at the tertiles of the event time distribution) and two boundary knots

$$\log(\lambda_{0}\left(t\right))=\gamma_{0}+\gamma_{1}B_{1}(t)+\gamma_{2}B_{2}(t)+\gamma_{3}B_{3}(t)$$

where the spline basis functions $B_{i}\left(t\right),\;i=1\text{,}2,3,$ are derived from the knots.^51^

Time-dependent effect of each variable is included using an interaction between each variable and the logarithm of time since diagnosis. As an example, the equation of the model is as follows, given two prognostic variables $x_{1}$, continuous, and $x_{2}$, categorical (with J categories, j=1,…J)

$$\lambda_{E}\;\left(t,x\right)=\lambda_{0}\left(t\right)*\exp(\beta_{1}(t)*f(x_{1})+\sum_{j=2}^{J}\beta_{2,j}(t)*I_{x_{2}=j})$$

where $f\left(x_{1}\right)=x_{1}$ if the effect of $x_{1}$ on the logarithm of excess mortality is linear, and $f\left(x_{1}\right)$ is a spline function when the effect of $x_{1}$ is not linear, while $\beta_{1}\left(t\right)=\beta_{1}$ if the effect of $x_{1}$ is proportional, and $\beta_{1}\left(t\right)=\beta_{1}*\log(t)$ if not; the same applies to $\beta_{2,j}(t)$.

We use the Stata commands *stns*^52^ to implement the PP cohort and period approaches, and *strcs*^53^ for fitting the flexible parametric models.

### 3.3 Model selection

We present two specific model-selection algorithms here, but wish to highlight that any other sound algorithm could be used. We adapt *mfpigen*, the model-selection algorithm designed by Royston and Sauerbrei, including tests for interactions,^54^ and our adaptation of *mvrs*^14^ for interactions^13^ using the AIC^22^ and the BIC.^55^ AIC is one of the criteria designed to express the ‘distance’ between two models,^20^ that is an estimate of the distance between our model and the model that did generate the data. AIC is defined from the log-likelihood of the model, L and its number of parameters, p.

The log-likelihood of the excess hazard models fitted here is

$$L\left(\beta |x,\;\delta_{i},\;t_{i}\right)=\sum_{i=1}^{N}\delta_{i}\log\left\{\lambda_{P}\left(t_{i},z\right)+\lambda_{E}\left(t_{i},\beta ,x\right)\right\}+\log\left\{S_{E}(t_{i})\right\}$$

such that

$$\mathrm{AIC}=-2*L\left(\beta |x,\;\delta_{i},\;t_{i}\right)+2*p$$

AIC can be shown to be equivalent to a likelihood ratio test multiplied by a constant, meaning that there is an associated positive probability (*p*-value) that it rejects the null hypothesis, when it is true. That *p*-value is 0.157 when models are nested and differing by 1*df*.^56,57^

BIC comes from a consistent class of criteria. It does not estimate the distance between the true model and the model under consideration, but aims to consistently point to the true model even when sample size increases, if the true model is part of the models considered. Its value varies with the number of parameters p and the number of events d.

$$\mathrm{BIC}=-2*L\left(\beta |x,\;\delta_{i},\;t_{i}\right)+p*\log(d)$$

The Royston and Sauerbrei algorithm is a succession of likelihood ratio tests comparing two models at a time in a logical sequential order. The algorithm starts by fitting the simplest model to the data, using linear and proportional effects of all variables. Starting with the most significant effect (i.e. lowest *p*-value), more complex versions of the effects of each variable are tested, one at a time, such as non-linearity and time-dependency.

Our adaptation follows the same logical st.tif, but the models’ AICs or BICs are compared, two at a time. If the lowest criterion is over two digits away from the larger criterion, the model pertaining to the larger criterion is discarded. If both models have criteria within two of each other, both models are kept, and more complex models derived from each of these are further compared. A rational for the choice of a difference of 2 is provided in section 3.4 using evidence ratios.

The original Royston and Sauerbrei algorithm yields one single model, from which all inference about measure of effects, associations, and outcome prediction is derived. Our proposed algorithm based on Information Criteria leads to the selection of several models, which are equally likely to have generated the data, given their AIC or BIC are within 2 digits of the minimum AIC or BIC.

### 3.4 Multi-model inference (model averaging)

In the following, XIC is used to stand for AIC or BIC, interchangeably. From the multiple models selected (i.e. models having similar support from the data), say M, we need to combine the M estimates to obtain one estimate of the excess hazard from which we derive the cohort cancer survival. Since the models are selected using XIC, each has a known XIC from which we derive XIC-weights as follows:

Let us define the model with lowest XIC (${\mathrm{XIC}}_{\min}$) as $m_{\min}$. We define the distance between $m_{\min}$ and any other model m, $\Delta_{m}={\mathrm{XIC}}_{m}-{\mathrm{XIC}}_{\min}$, and the likelihood of model m given the data is $M\left(m|x\right)=\exp(-\frac{1}{2}*\Delta_{m})$.^20^

The weights $w_{m}$ of each of the M models m reflect how much evidence there is for model m being the actual model that generated the data. Weights are defined such that they sum to 1, $\sum_{m=1}^{M}w_{m}=1$

$$w_{m}=\frac{M\left(m|x\right)}{\sum_{m=1}^{M}M\left(m|x\right)}$$

‘Evidence ratios’ for a model m versus model n are defined as the ratio of their weights $w_{m}$ and $w_{n}$, as

$$e\left(m,n\right)=\frac{w_{m}}{w_{n}}=\frac{\exp(-\frac{1}{2}*\Delta_{m})}{\exp(-\frac{1}{2}*\Delta_{n})}$$

If we suppose model m is the model with minimum XIC, we have

$$e\left(m,n\right)=\;\frac{1}{\exp(-\frac{1}{2}*\Delta_{n})}=\exp(\frac{1}{2}*\Delta_{n})$$

Therefore, we see exponential increase in evidence for the model with minimal XIC with increased distance to that XIC. The evidence ratio between models m and n is 2.7 if $\Delta_{n}=2$ (and 7.4 and 54.6 when $\Delta_{n}=4$ or 8, respectively). This is a rational for selecting models with XIC within two digits of the minimum XIC, where the evidence for m versus n is not so strong.

Given the potential complexity of the effects on the excess mortality hazard, we average the quantity modelled rather than the parameter estimates.^20^ This is specifically advised in Burnham and Anderson: “Structural parameters in non-linear models should not be averaged” and model averaging should rather be done on “the predicted expected response variable $\hat{E}(y)$”.^20^ Therefore, the XIC-weights are used to combine the model-based individual excess hazards estimated at each time t after diagnosis. Borrowing from the reasoning of both the algorithmic model-selection^16,58^ and the multi-model inference literature, we follow the st.tif below to combine the model-based estimates into a model-averaged estimate:

- Run XIC-based algorithm (e.g. *mfpigen* or the adapted *mvrs*)
- Isolate the M XIC-best models
- Calculate the XIC-weights, $w_{m}$, for each model m, (m=1,…,M)
- From the estimated parameters, derive the excess mortality hazards, at pre-defined times t after diagnosis (e.g. monthly) for each model m:$\;{\hat{\lambda }}_{i,m}(t)$ for each individual i with covariates $x_{i}$ in the data.
- Calculate the model-average excess mortality hazard (for patient i) at each pre-defined time t, such that
  $${\hat{\lambda }}_{i,MA}\left(t\right)=\;\sum_{m=1}^{M}{w_{m}*\hat{\lambda }}_{i,m}\left(t\right)$$

The model-average cumulative excess hazard, ${\hat{\Lambda }}_{i,MA}$ may easily be obtained as well

$${\hat{\Lambda }}_{i,MA}\left(t\right)=\;\sum_{m=1}^{M}{w_{m}*\hat{\Lambda }}_{i,m}\left(t\right)$$

f. If the quantity of interest is cohort net survival, we first calculate individual model-averaged net survival, ${\hat{S}}_{i,MA}\left(t\right)=\exp(-{\hat{\Lambda }}_{i,MA}\left(t\right))$ at each time t. Then, we estimate cohort net survival by averaging the individual net survival values, ${\hat{S}}_{MA}(t)=\frac{1}{N}\sum_{i=1}^{N}{\hat{S}}_{i,MA}(t)$ at time t.

The unconditional variance estimator of the model-averaged estimate is derived in Burnham and Anderson (pp.158–164)^20^ and follows earlier work presented in Buckland et al.^17^ We adapted this derivation to our setting where we averaged the predicted expected response variable (i.e. the excess mortality hazards). The variance estimator for the model-averaged outcome combines the XIC-weights, $w_{m}$, and the estimated variances of each individual model estimates, $\hat{\mathrm{var}}({\hat{\lambda }}_{i,m}(t))$, such that

$$\hat{\mathrm{var}}\left({\hat{\lambda }}_{i,MA}\left(t\right)\right)={\left\{\sum_{m=1}^{M}w_{m}*\sqrt{\hat{\mathrm{var}}({\hat{\lambda }}_{i,m}(t))+{\left({\hat{\lambda }}_{i,m}(t)-{\hat{\lambda }}_{i,MA}\left(t\right)\right)}^{2}}\right\}}^{2}$$

This estimator has components of within-variation ($\hat{\mathrm{var}}({\hat{\lambda }}_{i,m}(t))$) and between variation ${\left({\hat{\lambda }}_{i,m}(t)-{\hat{\lambda }}_{i,MA}\left(t\right)\right)}^{2}$, thus quantifying the uncertainty with regards to model selection. This unconditional variance estimator assumes perfect pairwise correlation between ${\hat{\lambda }}_{i,m}(t)-{\hat{\lambda }}_{i,MA}\left(t\right)$ and ${\hat{\lambda }}_{i,n}\left(t\right)-{\hat{\lambda }}_{i,MA}\left(t\right)$, as derived from models m and n. This leads to a conservative variance estimate, i.e. the estimated variance tends to be too large.^17^

### 3.5 Checking predictions

After selecting a (or a set of) best model(s), predicting our outcome of interest, and averaging the outcomes, we are interested in quantifying the distance between these estimates and the observed cancer survival of the patients. Since we have follow-up information until 31 December 2015, we estimate net survival of patients diagnosed in 2010 and 2011 using the PP non-parametric estimator of cancer survival (see section 3.2. above).

We aim to compare our predictions to what will be estimated in the future, given the data available then. We recognise that the PP estimator of survival, often used for policy making and planning, is a consistent estimator of net survival, but cannot be regarded as the ‘truth’.

To quantify the difference between the population-based prediction using our model-average estimate and the PP net survival estimates, we define the Root Mean Integrated Square Difference (RMISD) of prediction. This measure contrasts the predicted survival to the estimated PP survival and we approximate this quantity using G groups defined by age group and deprivation quintile (low-resolution data) or by age group, stage and deprivation quintile (high-resolution data)

$$\mathrm{RMISD}=\sqrt{\frac{1}{G}\sum_{g=1}^{G}\int {\left({\hat{S}}_{g}\left(u\right)-S_{g}(u)\right)}^{2}du}$$

$S_{g}\;$ is the non-parametric PP estimate of cancer survival for group g, while ${\hat{S}}_{g}$ is the prediction of survival for the same group of patients using (i) model-averaging, or (ii) a pre-selected simple model or (iii) the period approach. The integral is approximated using the Gauss-Legendre quadrature with 20 nodes. We choose to calculate RMISD for survival measured at one and five years after diagnosis.

## 4 Results

### 4.1 Low-resolution data setting: empirical evaluation of the properties of multi-model inference

#### 4.1.1 Description of the data

Between 1990 and 2010, there were an average of 18,233 and 8,636 men diagnosed with lung and colon cancer, respectively, and 32,493 women diagnosed with breast cancer, every year. The number of cancer patients was multiplied by at least 1.5 for breast (women) and colon (men), but slightly decreased for lung cancer (men). Five-year net survival increased for all cancers between 1990 and 2010, with the largest increase for lung cancer, from 5.3% in 1990 to 9.0% in 2010 (online Appendix Table 1).

**Table 1.** Models selected following model selection algorithms, by cancer and cohort of patients used in model selection.

									RMISD at 5 years	
	* *	Age (A)	Year of diagnosis (Y)	Deprivation (D)	Interactions	XIC^a^	XIC^a^ weights (%)	XIC^a^ distance with next selected model	2010^b^	2011^c^
Breast cancer										
*Algorithm adapted for interactions*										
AIC	2005–2010	NL TD	NL TD	TD	A*D (TD), Y*D	205,554.5		18	0.030	0.028
	2000–2010	NL TD	NL TD	TD	A*D (TD), Y*D	589,478.3		61	0.032	0.032
	1995–2010	NL TD	NL TD	TD	A*D (TD), Y*D (TD)	1,072,626.0	69		0.032	0.033
	1995–2010	NL TD	NL TD	TD	A*D (TD), Y*D	1,072,628.0	31	104		
	1990–2010	NL TD	NL TD	TD	A*D (TD), Y*D (TD)	1,605,374.0		12	0.039	0.042
BIC	2005–2010	NL TD	NL TD	PH		205,742.0		18.3	0.026	0.026
	2000–2010	NL TD	NL TD	TD	Y*D	589,840.6		10.4	0.033	0.032
	1995–2010	NL TD	NL TD	TD	Y*D	1,073,076.0		406.0	0.033	0.033
	1990–2010	NL TD	NL TD	TD	Y*D (TD)	1,605,849.0		53.4	0.039	0.042
*mfpigen algorithm*										
AIC	2005–2010	NL TD	NL TD	TD	A*D	205,576.9			0.024	0.027
	2000–2010	NL TD	NL TD	TD	A*D	589,540.4			0.031	0.034
	1995–2010	NL TD	NL TD	TD	A*D	1,072,766.0			0.030	0.035
	1990–2010	NL TD	NL TD	TD	A*D	1,605,607.0			0.039	0.045
BIC	2005–2010	NL TD	NL TD	PH		205,742.0		18.3	0.026	0.026
	2000–2010	NL TD	NL TD	TD		589,766.8		8.6	0.031	0.033
	1995–2010	NL TD	NL TD	TD		1,073,035.0		13.8	0.030	0.034
	1990–2010	NL TD	NL TD	TD	A*D	1,605,901.0		9.5	0.039	0.045
*Simple models* ^d^										
	2005–2010	L PH	L PH	PH		208,763.6			0.069	0.075
	2000–2010	L PH	L PH	PH		598,469.7			0.079	0.087
	1995–2010	L PH	L PH	PH		1,087,460.8			0.088	0.098
	1990–2010	L PH	L PH	PH		1,625,430.8			0.089	0.098
*Period approach*										
Colon cancer										
*Algorithm adapted for interactions*										
AIC	2005–2010	NL TD	L TD	TD		97,225.4	65		0.094	0.117
	2005–2010	NL TD	NLTD	TD		97,226.7	35	4		
	2000–2010	NL TD	NL TD	TD	A*D (TD), Y*D	215,214.2		39	0.097	0.110
	1995–2010	NL TD	NL TD	TD	Y*D	338,571.2		192	0.093	0.108
	1990–2010	NL TD	NL TD	TD	A*D (TD), Y*D	455,678.2		85	0.095	0.110
BIC	2005–2010	NL TD	NL TD	PH		97,379.7		637.0	0.095	0.116
	2000–2010	NL TD	NL TD	TD		215,438.8		1,483.9	0.093	0.108
	1995–2010	NL TD	NL TD	TD		338,747.7		171.3	0.091	0.109
	1990–2010	NL TD	NL TD	TD		455,968.5		3,315.6	0.093	0.111
*mfpigen algorithm*										
AIC	2005–2010	NL TD	NL TD	TD	A*D	97,225.2	67		0.090	0.115
	2005–2010	NL TD	NL TD	TD		97,226.7	33			
	2000–2010	NL TD	NL TD	TD	A*D	215,253.6			0.094	0.111
	1995–2010	NL TD	NL TD	TD	A*D	338,556.8			0.090	0.109
	1990–2010	NL TD	NL TD	TD	A*D	455,763.4			0.092	0.112
BIC	2005–2010	NL TD	NL TD	PH		97,379.7		67.3	0.095	0.116
	2000–2010	NL TD	NL TD	TD		215,438.8		41.2	0.093	0.108
	1995–2010	NL TD	NL TD	TD		338,747.7		72.7	0.091	0.109
	1990–2010	NL TD	NL TD	TD		455,968.5		66.9	0.093	0.111
*Simple models* ^d^										
	2005–2010	L PH	L PH	PH		98,608.6			0.175	0.170
	2000–2010	L PH	L PH	PH		217,925.7			0.155	0.150
	1995–2010	L PH	L PH	PH		342,308.5			0.147	0.138
	1990–2010	L PH	L PH	PH		460,688.7			0.141	0.138
*Period approach*										
Lung cancer										
*Algorithm adapted for interactions*										
AIC	2005–2010	NL TD	NL TD	TD	Y*D	110,543.9		410	0.122	0.142
	2000–2010	NL TD	NL TD	TD	A*D, Y*D	220,096.1		31	0.103	0.128
	1995–2010	NL TD	NL TD	TD	A*D, Y*D	323,089.4		1,041	0.102	0.127
	1990–2010	NL TD	NL TD	TD	A*D, Y*D	418,484.9		1,386	0.102	0.126
BIC	2005–2010	NL TD	NL TD	PH		101,805.6		573.1	0.120	0.148
	2000–2010	NL TD	NL	PH		202,668.9		932.1	0.107	0.125
	1995–2010	NL TD	NL TD	PH		290,457.4		423.2	0.105	0.129
	1990–2010	NL TD	NL TD	TD		378,957.9		193.5	0.105	0.127
*mfpigen algorithm*										
AIC	2005–2010	NL TD	NL TD	TD	A*D	101,644.7			0.112	0.147
	2000–2010	NL TD	NL TD	TD	A*D	202,427.4			0.102	0.128
	1995–2010	NL TD	NL TD	TD	A*D	290,194.5			0.101	0.125
	1990–2010	NL TD	NL TD	TD	A*D	378,710.8			0.100	0.126
BIC	2005–2010	NL TD	NL TD	PH		101,805.6		69.8	0.120	0.148
	2000–2010	NL TD	NL	PH		202,668.9		75.6	0.107	0.125
	1995–2010	NL TD	NL TD	PH		290,457.4		63.1	0.105	0.129
	1990–2010	NL TD	NL TD	TD		378,957.9		55.0	0.105	0.127
*Simple models* ^d^										
	2005–2010	L PH	L PH	PH		102,492.2			0.119	0.113
	2000–2010	L PH	L PH	PH		203,852.5			0.119	0.113
	1995–2010	L PH	L PH	PH		292,678.4			0.125	0.117
	1990–2010	L PH	L PH	PH		381,722.1			0.124	0.117
*Period approach*									0.076	

L: Linear; NL: non-linear; TD: time-dependent; PH: proportional hazard; *: interaction; AIC: Akaike information criteria; BIC: Bayesian or Schwarz information criteria.

^a^XIC stands for AIC or BIC depending on model.

^c^RMISD are calculated by averaging the integrated square differences measured in each of the age and deprivation groups.

^d^Prediction of five-year survival for patients diagnosed in 2010 and 2011.

^d^A model with all variables modelled with a linear proportional effect.

#### 4.1.2 Model selection

The functional forms of the selected variables are displayed in Table 1 (columns 1-4), along with the XIC of the selected model(s) (column 5), the XIC-distance to the model with the closest XIC (column 7), and where appropriate, XIC-weights (column 6). Adding earlier cohorts to patients diagnosed in 2005–2010 hardly changes the functional form selected for the effects of age, year of diagnosis or deprivation, as well as their interactions, especially when using the *mfpigen* algorithm with AIC, or when using BIC (with either algorithm). More complex models (including time-dependent effects of the interaction between age and deprivation) are selected by our adapted algorithm using AIC: these include time-dependent age-deprivation interactions (breast cancer) and age-deprivation and year-deprivation interactions (lung cancer). With BIC selection, there is almost no difference in the complexity of the models selected by *mfpigen* and our adapted algorithm: the models selected are identical for colon and lung cancers, and the only selected interactions differ for breast cancer. To contrast with the models selected, we also apply a simple model with all variables modelled with a linear (when continuous), proportional hazard effect on excess mortality, in each of the four cohorts of patients. The XIC values of these simple models (Table 1) are constantly higher than that of the selected models except for the AIC values of the lung cancer models selected by our adapted algorithm.

#### 4.1.3 Root mean integrated square difference for the prediction of net survival

**Figure 2.**
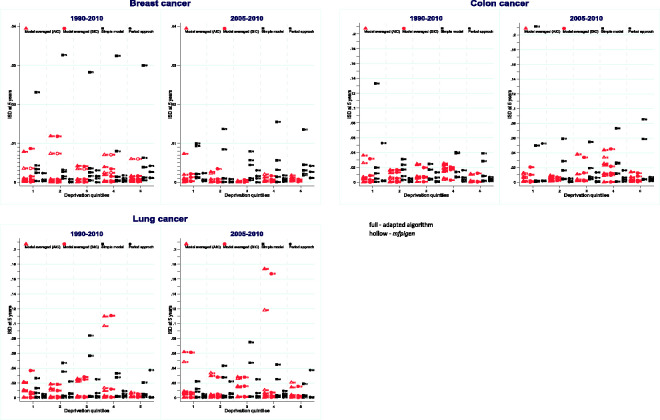
Integrated Square Difference (ISD) between NS predicted by each AIC or BIC model-averaged, simple models, and the period approach, compared to the PP cohort survival for patients diagnosed in 2010, by age group, deprivation from 1990 to 2010 and from 2005 to 2010 cohorts of patients used in model selection.

Root mean integrated square difference (RMISD) is measured throughout the first five years after diagnosis. By group defined by age and deprivation level, we calculated Integrated Square Differences (ISD) (see formula of the RMISD in section 3.5.) between model-averaged net survival estimates and the PP estimates using known follow-up until 31 December 2015 for patients diagnosed in 2010 (Figure 2 for the cohorts 1990–2010 and 2005–2010, and online Appendix Figure 1 for all four cohorts), and for patients diagnosed in 2011 (online Appendix Figure 2).

*Breast cancer*: All ISDs are very small, and largest differences are seen for the oldest age-group when survival is predicted by the simple model. Similar observations can be made for the projection of survival for patients diagnosed in 2011, not included in model selection (online Appendix Figure 2). The more recent the cohorts of patients, the better the estimates of survival: ISDs are smaller when using 2005–2010 cohorts only versus 1990–2010 cohorts.

*Colon cancer*: Simple models lead to high ISD for different age and deprivation groups, such as patients aged 15–54 years in the most deprived group and 45–54 years in deprivation quintile 4. Except for patients aged 15–44 in the least deprived group, 2010-period approach estimates show low ISD. ISD values remain stable and low, whatever the number of cohorts used in multivariable model-averaged prediction of survival (online Appendix Figure 1). ISDs for patients aged 15–44 and 45–54 years are slightly higher when the models are used for projection of survival for patients diagnosed in 2011.

*Lung cancer*: Except for patients aged 15–44, in deprivation quintile 4, for whom model-averaged ISD is large, model-averaged ISD are generally lower than ISD derived from the simple model, and smaller or similar to most of the 2010-period ISDs. Model-averaged predictions for patients aged 15–44, in deprivation quintile 3 and diagnosed in 2011 show very high ISD. Such large ISDs are also observed, but to a lesser extent, for simple model estimates (online Appendix Figure 2).

The highlighted patterns in ISD, for all three cancers, are observed for (i) AIC (triangular shapes) and BIC (circular shapes) selected models, and following (ii) model selection using *mfpigen* (hollow red symbols) and our adapted algorithm (full red symbols).

By averaging the ISD values displayed in Figure 1 and online Appendix Figures 1 and 2, the RMISD values summarise the overall differences in the survival curves (Table 1). For all cancers, model-averaged estimates of survival lead to the smallest RMISD, in comparison to using pre-defined simple models (Table 1). Nonetheless, there are differences between cancers: for breast cancer, there is a small advantage in restricting the model selection and estimation to the cohorts of patients diagnosed in the last five years, while for patients diagnosed with colon or lung cancer, longer time-trends yield better estimates of survival for patients for whom follow-up is not yet available. The simple models yield the highest RMISD values, for each cancer and each cohort, except for lung cancer when using the AIC-based multi-model inference from the adapted *mvrs*. (Table 1)

**Figure 3.**
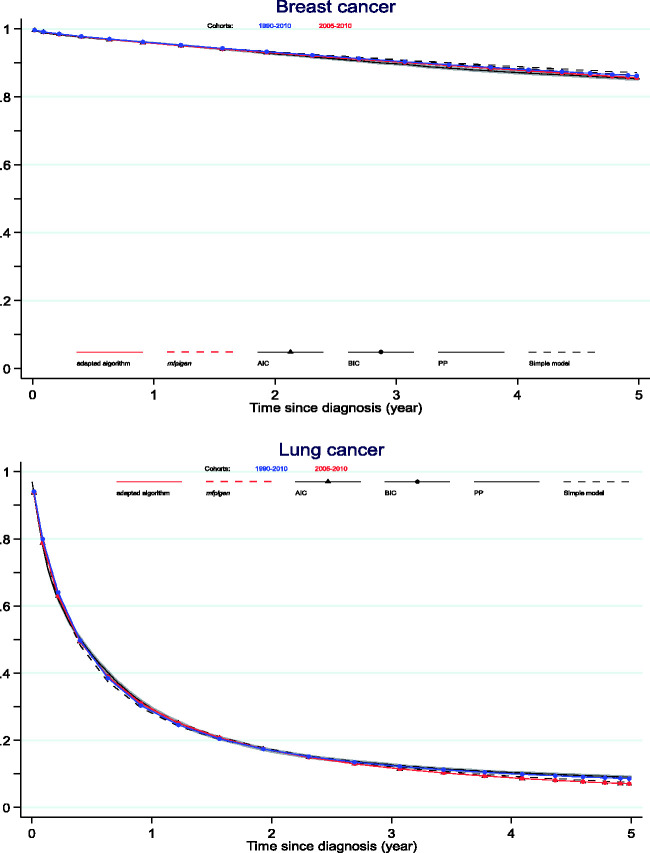

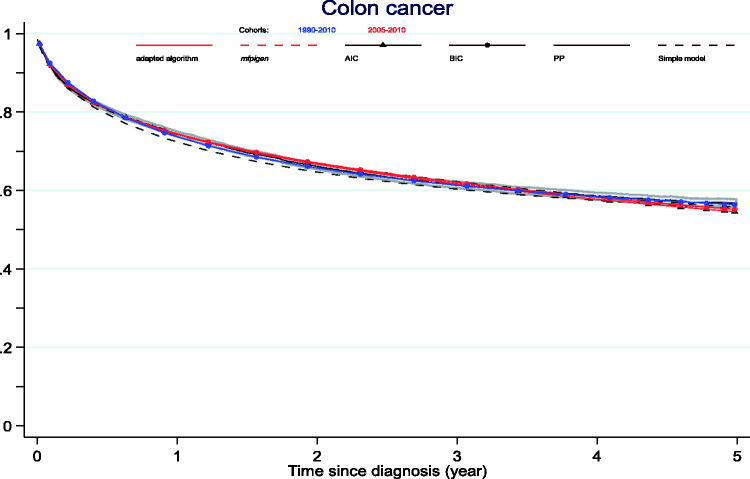
Net survival curves: comparison between the PP estimates and estimates from 1990 to 2010 and from 2005 to 2010 cohorts of patients used in model-averaging from AIC and BIC model selection.

Figure 3 shows what the actual differences are on the overall cohort net survival curves, contrasting cancer survival estimated from the simple model, and from model-averaged selection, to the 2010-cohort approach. The differences between the model-averaged estimates of survival up to five years are tiny when contrasting AIC and BIC selection, adapted *mvrs* or *mfpigen* algorithm. Nonetheless, they do reflect the conclusions from RMISD: additional cohorts of patients are necessary for a better prediction of lung cancer survival. Net survival estimated from model selection and when necessary, model averaging, are closer to the PP estimates than estimates from simple models.

### 4.2 High-resolution data setting: illustration

This illustration rests on richer datasets to allow inclusion of the effects of potentially key prognostic factors such as stage at diagnosis, mode of presentation (emergency presentation for lung cancer, screening for breast cancer) and performance status (lung cancer) on the excess hazard.

For both cancers, between 1 and 10 models have similar support from the data, given their AIC, but only one model given its BIC, when restricting to models with BICs within two of each other: we report the effects estimated, the AIC or BIC and corresponding weights in Table 2. The models selected to model breast cancer survival have AIC weights between 16.1% and 42.3% (adapted *mvrs*) and between 6.6% and 17.8% (*mfpigen*); the models selected to model lung cancer survival have AIC weights between 11.6% and 29.4% (adapted *mvrs*). The selected models with the highest AICs are only just over two units away from the next model: 2.2 for breast (both algorithms) and lung (*mfpigen*), but 316.3 units away for lung (adapted *mvrs*). The effects of deprivation (PH) and stage (TD) for breast cancer, and the effects of stage (TD), performance status (TD), emergency presentation (TD), and an interaction between age and deprivation for lung cancer, are selected in all models.

**Table 2.** Models selected, and associated AIC, BIC for the prediction and projection of four-year cancer survival.

	Age (A)	Year of diagnosis (Y)	Deprivation (D)	Stage (S)	Performance status (PS, lung)	Presentation: screening (breast) emergency (lung)	Interactions	XIC^b^	XIC^b^ weights (%)	XIC^b^ distance with next selected model
*Breast cancer*										
(adapted)	L TD	NL TD	PH	TD		PH		9,481.6	23.4	
	L TD	NL	PH	TD		PH		9,480.4	42.3	
	L TD	NL TD	PH	TD		TD		9,482.1	18.2	
	L	NL	PH	TD		TD		9,482.4	16.1	2.2
(mfpigen)	L TD	NL	PH	TD		PH		9,480.4	17.8	
	NL TD	L	PH	TD		PH	A*D	9,481.1	13.1	
	NL TD	L	PH	TD		PH		9,480.7	15.7	
	L TD	NL TD	PH	TD		PH		9,481.6	9.9	
	NL	L	PH	TD		TD	A*D	9,482.1	7.8	
	NL	L	PH	TD		TD		9,482.1	7.7	
	L TD	NL TD	PH	TD		TD		9,482.1	7.7	
	L	NL	PH	TD		TD		9,482.4	6.8	
	NL	L	PH	TD		PH	A*D	9,482.3	7.0	
	NL	L	PH	TD		PH		9,482.4	6.6	2.2
BIC-selection (adapted & mfpigen)	L	NL	PH	TD		PH		9,576.3^c^		
Simple model^a^	L	L	PH	PH		PH		9,619.2		
*Lung cancer*										
(adapted)	L	NL	TD	TD	TD	TD	A*D	7,820.2	15.5	
	L	NL TD	PH	TD	TD	TD	A*D	7,819.0	29.4	
	L	NL	PH	TD	TD	TD	A*D	7,819.1	26.8	
	L	NL TD	TD	TD	TD	TD	A*D	7,820.1	16.6	
	L TD	NL TD	PH	TD	TD	TD	A*D	7,820.8	11.6	316.3
(mfpigen)	NL	L TD	PH	TD	TD	TD	A*D	7,819.9		2.2
BIC-selection (adapted & mfpigen)	L	NL	PH	TD	TD	TD		7,967.4^c^		
Simple model^a^	L	L	PH	PH	PH	PH		8,369.8		

L: Linear; NL: non-linear; TD: time-dependent; PH: proportional hazard; *: interaction; AIC: Akaike information criteria; BIC: Bayesian or Schwarz information criteria.

^a^A model with all variables modelled with a linear proportional effect.

^b^XIC stands for AIC or BIC depending on model.

^c^BIC value.

Model-averaged estimates of the excess hazard are presented in online Appendix Figure 3, highlighting differences between these and those estimated by simple models, especially for stage IV with larger excess hazard estimated with the simple models.

**Figure 4.**
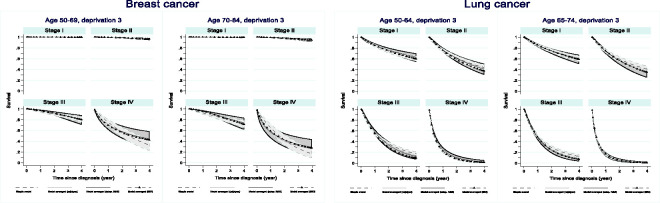
Up to four-year net survival for patients diagnosed with breast or lung cancer by age and stage for patients in the third deprivation quintile.

There is very little difference between the AIC (*mfpigen* and adapted *mvrs*) and BIC model-averaged survival curves for patients diagnosed with breast or lung cancer (Figure 4). Survival estimated from the simple model, although modelling the effects of all variables, does differ for both cancers, especially at stages IV (breast) and II and III (lung).

The confidence intervals around the net survival curves highlight uncertainty related to data sparsity but also model selection.

## 5 Discussion

We contrast the predictions and projections of cancer survival derived from a model-averaged approach and (i) a-priory simple model and (ii) non-parametric period approach. We use an algorithm for model selection that has been used in cancer epidemiology,^13^ for scanning methodically through the possible effects of independent factors on the excess hazard of death, merely to illustrate the multi-model inference in cancer survival. Indeed, any other algorithm based on screening through possible effects could have been adapted to the information criteria paradigm. We implement the model averaging methodology for the selection of the best model(s)^20^ using AIC and BIC as selection criteria. We show the lethality and the rate of improvement of cancer survival determine how many past cohorts of patients are needed to predict and project survival with best accuracy. We also show that allowing for multi-model estimation of cancer survival generally results in restricted mean integrated square differences as good as or better than the non-parametric period approach. In some cases, despite larger AICs or BICs, simple models produced accurate predictions, similar to model-averaged predictions, but projections from these models do not estimate cancer survival as well.

There are many advantages to estimating survival using IC-based model selection and multi-model inference. (1) Transparent model building strategy: the algorithm walks through the effects of variables in a hierarchical and systematic fashion. (2) Uncertainty relative to model selection is taken into account in the variance of the estimated outcomes. (3) There is no assumption that an effect is simple, without checking it can or needs to be simple. (4) Projections for patients outside of the training sample are possible, which is not possible using the period approach.

The results show that for breast cancer patients, only patients diagnosed in the five years prior to the year for which we need to make five-year survival predictions are needed to produce accurate predictions and projections. This can be explained by survival increasing at constant pace of about 3–8% per five years in the last 20 years. By contrast, for lung and colon cancers, cancer survival increased irregularly in the last 20 years: close to 30% increase in five-year lung cancer survival between 2005 and 2010, but no increase between 1990 and 1995 and similarly, 12% increase in colon cancer survival between 2005 and 2010 but only 3% between 2000 and 2005. More cohorts of patients are needed to predict and project five-year survival accurately, due to these irregular trends in survival. These considerations need to be borne in mind when using the most recent cohorts for the prediction of cancer survival.

Bayesian, cross-validation and bootstrap-based approaches are also likely to perform well in excess hazard model selection. Nonetheless these carry high computational demands. BIC readily links with Bayesian model averaging and is asymptotically consistent in estimating the true generating model.^59^ Despite AIC asymptotically equivalent to cross-validation,^23^ and therefore a tool of choice for model selection in the context of prediction, it tends to overestimate the dimension of the true model.^59^ Furthermore, multi-model inference has theoretical and practical advantages, particularly for predictions.^20^ These advantages include: (1) taking into account uncertainty in model selection, leading to more robust results whowho do not necessarily depend upon a particular model; (2) choosing to average models that have AIC within two of the minimum AIC helps keep the number of considered models reasonable; (3) model averaging avoids one to have to defend the choice of model: it makes convincing stakeholders from different backgrounds and highlighting the robustness of the results easier.^60^ We recognise the limitation that model uncertainty remains conditional on the model set, as all models come from a unique model set.^61^ Other approaches which have proved useful for predictions would broaden the model sets considered (e.g. LASSO, Random Survival Forest) and could provide interesting research developments but would need to be adapted to the relative survival data setting.

We focus here on predicting and projecting five-year net survival, as most events happen in the short term following a cancer diagnosis, certainly for colon and lung cancers. By contrast, breast cancer patients experience long-term excess mortality. Therefore, we performed additional analyses for the prediction of 10-year breast cancer survival. We found that a model-averaged 10-year survival prediction leads to a smaller difference than from a simple model or from a non-parametric period approach (data not shown).

Using empirical data in the low-resolution setting, we can only compare our predictions and projections to the consistent non-parametric PP estimates of cancer survival, for patients diagnosed in a given year. We acknowledge that these remain estimates of survival, rather than the “truth”, but we argue that they will be what is produced when the follow-up information becomes available, to contrast trends in cancer outcomes.^62–64^ Nonetheless, both non-parametric and parametric outcomes are estimating the same quantity since the models are adjusting for the variables that constitute the strata of the PP estimates.

However, in the application, due to data sparsity by strata defined by the values of the variables adjusted for in the models, it was not possible to compare the model-averaged estimates of net survival to the PP. Indeed, when the PP is not stratified by the same prognostic factors, it is not estimating the same quantity as the model-based estimates. The results of the high-resolution setting are presented to motivate the use of multi-model inference for the prediction and projection of cancer survival. The differences between predictions derived from a simple model versus IC-based approach, however, highlight that it would be relevant to conduct such comparison in a larger population in which variables such as stage at diagnosis, mode of presentation and performance status are available.

Multi-model inference, as presented here, allows model parameters to remain the raw information for the estimation of each model’s outcome of interest. Such outcomes are then averaged, and interpretation of the predictions can only be made on the outcome. Multi-model inference increases the ability to perform better predictions while retaining interpretability of the averaged outcomes.^61,65^ It seems to be a good compromise between best-model selection strategy (high interpretability but poor predictions) and ensemble learning strategy (high predictions but poor interpretability). For patients and their carers, prediction of the remaining survival time represents their main interest. However, this point estimate of time carries poor predictive capability.^9^ Hence, much of the literature focuses on prediction of survival probabilities, at individual or population level. In the field of prognosis research at individual level, there is a growing emphasis on improving the quality of published risk scores so they are useful to individual patient prognosis.^12^

Here, we aim to predict and project population-based levels of survival, rather than individual cancer survival predictions. It is the reason why we do not rely on standard loss functions, or usual measures of discrimination and calibration. It is still important to gather accurate information on the main prognostic factors, and make sure models are correctly specified since correct model specification and availability of individual patient characteristics improve prediction. All of this is exemplified in both scenarios here, low- and high-resolution data settings, in which complex prognosis models are compared.

## 6 Conclusion

We recommend that, given a set of variables that may influence levels of cancer mortality, possible excess hazard models should be assessed systematically. We encourage analysts to consider that a model may not be singled out as the best model. Model averaging using Kullback-Leibler distance such as AIC, or Bayesian principles such as BIC, allows users to consider several equivalent models and effects, and to take account of the uncertainty relative to model selection in the estimation of the variance of the outcomes. Prediction and projection of cancer survival can best be done using such carefully selected parametric models.

## Supplemental Material

sj-xlsx-1-smm-10.1177_0962280220934501 - Supplemental material for Prediction of cancer survival for cohorts of patients most recently diagnosed using multi-model inference Click here for additional data file. Supplemental material, sj-xlsx-1-smm-10.1177_0962280220934501 for Prediction of cancer survival for cohorts of patients most recently diagnosed using multi-model inference by Camille Maringe, Aurélien Belot and Bernard Rachet in Statistical Methods in Medical Research

sj-xlsx-2-smm-10.1177_0962280220934501 - Supplemental material for Prediction of cancer survival for cohorts of patients most recently diagnosed using multi-model inference Click here for additional data file. Supplemental material, sj-xlsx-2-smm-10.1177_0962280220934501 for Prediction of cancer survival for cohorts of patients most recently diagnosed using multi-model inference by Camille Maringe, Aurélien Belot and Bernard Rachet in Statistical Methods in Medical Research

sj-xlsx-3-smm-10.1177_0962280220934501 - Supplemental material for Prediction of cancer survival for cohorts of patients most recently diagnosed using multi-model inference Click here for additional data file. Supplemental material, sj-xlsx-3-smm-10.1177_0962280220934501 for Prediction of cancer survival for cohorts of patients most recently diagnosed using multi-model inference by Camille Maringe, Aurélien Belot and Bernard Rachet in Statistical Methods in Medical Research

sj-xlsx-4-smm-10.1177_0962280220934501 - Supplemental material for Prediction of cancer survival for cohorts of patients most recently diagnosed using multi-model inference Click here for additional data file. Supplemental material, sj-xlsx-4-smm-10.1177_0962280220934501 for Prediction of cancer survival for cohorts of patients most recently diagnosed using multi-model inference by Camille Maringe, Aurélien Belot and Bernard Rachet in Statistical Methods in Medical Research
